# Supplementary material for: Spearmint (Mentha spicata L.) Phytochemical Profile: Impact of Pre/Post-Harvest Processing and Extractive Recovery
Source: Molecules. 2022 Mar 30;27(7):2243. doi: 10.3390/molecules27072243 (PMC9000270; doi:10.3390/molecules27072243)
Supplement: Supplementary file 1 [file molecules-27-02243-s001.zip › molecules-1637098-supplementary.pdf]

Supplementary Material

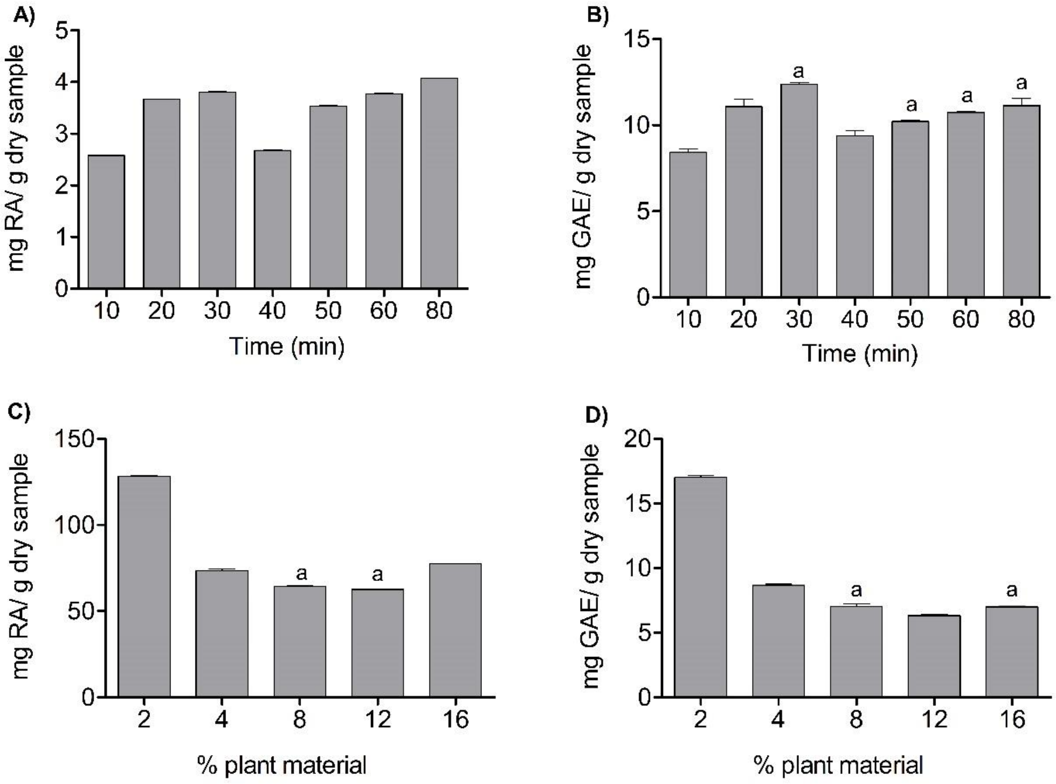

Figure S1. Extraction time selection and solid/solvent ratio selection. Rosmarinic acid (A and C) and content of total polyphenols (TPC) (B and D) in extracts obtained. The analysis statistic uses a one-way ANOVA (Bonferroni test, $p < 0.05$). Same letters indicate no there is a statistically significant difference.

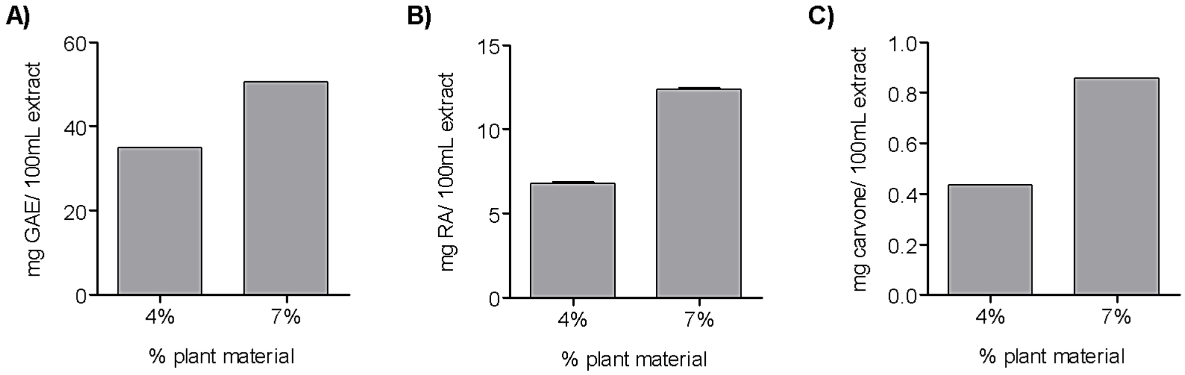

Figure S2. Solid/solvent extraction ratio. Total polyphenol content (TPC) (A); Rosmarinic acid (B) and Carvone (C) in extracts obtained with different proportions of material vegetable. Statistical analysis uses a one-way ANOVA (Bonferroni test, p < 0.05).

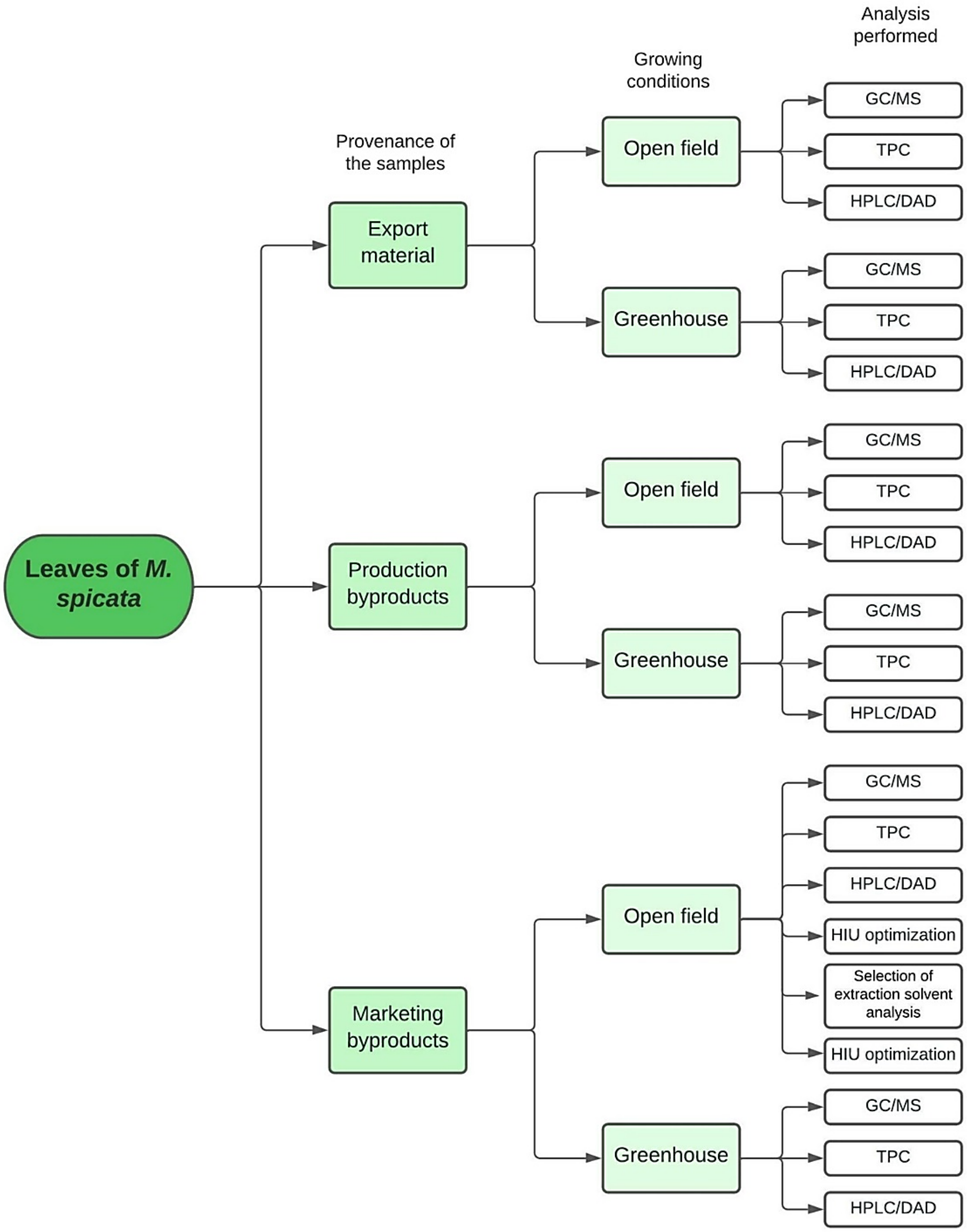

Figure S3. Summary of experimental treatments and analyses performed.

Table S1. Parameters of the second polynomial regression model for Rosmarinic acid

Factor	Sum of squares	DF	F-ratio	P-value	R^2
TPC					
Amplitude (X_1)	333.155	1	37.32	0.0000	
Time (X_2)	1209.57	1	135.50	0.0000	
X_1^2	51.6658	1	5.79	0.0254	0.92
X_2^2	191.02	1	21.40	0.0154	
X_1X_2	25.752	1	2.88	0.0001	

Table S2. Parameters of the second polynomial regression model for total content polyphenols

Factor	Sum of squares	DF	F-ratio	P-value	R^2
TPC					
Amplitude (X_1)	7608.37	1	79.89	0.0000	
Time (X_2)	8208.28	1	86.19	0.0000	
X_1^2	5415.07	1	56.86	0.0000	0.92
X_2^2	1059.56	1	11.13	0.0031	
X_1X_2	1303.34	1	13.69	0.0013	

Table S3. Parameters of the second polynomial regression model for carvone

Factor	Sum of squares	DF	F-ratio	P-value	R^2
TPC					
Amplitude (X_1)	0.4999	1	2438.83	0.0000	
Time (X_2)	0.6689	1	336.25	0.0000	
X_1^2	0.1204	1	587.44	0.0000	0.99
X_2^2	0.0043	1	21.17	0.0000	
X_1X_2	0.0056	1	27.57	0.0002	
